# Editorial: Immune-Modulatory Effects of Vitamin D

**DOI:** 10.3389/fimmu.2020.596611

**Published:** 2020-09-29

**Authors:** Susu M. Zughaier, Erik Lubberts, Abdulbari Bener

**Affiliations:** ^1^Department of Basic Medical Sciences, College of Medicine, QU Health, Qatar University, Doha, Qatar; ^2^Erasmus MC, University Medical Center Rotterdam, Rotterdam, Netherlands; ^3^Faculty of Medicine, Istanbul University Cerrahpasa, Istanbul, Turkey; ^4^International School of Medicine, Istanbul Medipol University, Istanbul, Turkey

**Keywords:** vitamin D, immune modulation, monocytes, T cells, infection

Vitamin D plays an essential role in bone development. However, recent studies are beginning to uncover its role as a modulator of the immune system (1). Several reports have shown associations between vitamin D deficiency (2) and the incidence as well as the severity of chronic inflammatory diseases such as cardiovascular disease (3, 4), inflammatory bowel disease (5), asthma (6), and chronic obstructive pulmonary disease (COPD) (7). Consistent with this, vitamin D supplementations have been shown to reduce the severity of and inflammation markers in chronic inflammatory diseases (8). At the molecular level, the hormonally active form of vitamin D (α1,25 dihydroxyvitamin D_3_) regulates the expression of vitamin D responsive genes that can lead to differential regulation of signaling pathways in immune cells. For example, vitamin D positively regulates iron homeostasis and erythropoiesis via the iron-hepcidin-ferroportin axis (9). Vitamin D deficiency is highly prevalent world-wide including countries with abundance of sunshine (10). Singh et al. reviewed the causes of vitamin D deficiency where they dissected the complex impact of genetic predisposition, gut microbiota, and immune system. In this review, authors examined GWAS database (11) and listed genes variants with SNPs that associate with risk of vitamin D deficiency. These alleles are common in vitamin D receptor (VDR) and vitamin D binding protein (VDPB). Since gut microbiota plays a crucial role in nutrients and vitamins production, absorption and degradation, authors also highlighted the role of vitamin D metabolism and VDR is regulating host-gut microbiota interactions.

At the cellular level, vitamin D exerts anti-inflammatory effects on immune cells that express the vitamin D receptor (VDR) such as monocytes, macrophages, and T lymphocytes, which in turn shapes the immune response during the onset of inflammation and infection and following vaccination. Vitamin D exerts anti-inflammatory effects by reducing pro-inflammatory cytokines production from macrophages and T cells (12, 13). Carlberg eloquently demonstrated that vitamin D/VDR signaling impacts chromatin modeling leading to significant modification of human monocytes epigenome during perturbation, consequently reducing cytokine release and modulating trained innate immunity. The immune modulatory effects of vitamin D include reduction in inflammatory cytokines release such as IL-1β, which is induced via inflammasome activation. Rao et al. showed that VDR inhibited NALP3-inflammasome activation leading to reduction in IL-1β release. To confirm the inhibitory effect of VDR on NLP3 inflammasome activation, authors used VDR-deficient mice and showed that IL-1β release was significantly reduced *in vivo* confirming that VDR inhibited NALP3 inflammasome activation. Vitamin D deficiency is associated with bone pain in particular and chronic disease pain in general (12, 14). The proposed mechanisms by which Vitamin D/VDR signaling modulate pain sensation is reviewed by Habib et al.. Authors delineate vitamin D/VDR interactions with pain sensing pathways including dorsal root ganglion (DRG) neurons, nerve growth factor (NGF), epidermal growth factor receptor (EGFR), glial-derived neurotrophic factor (GDNF), and opioid receptors.

Sufficient levels of vitamin D have been shown to regulate T cell proliferation by controlling T cell antigen receptor and T cell activation as well as enhancing the phagocytic activity of macrophages (12, 14). The immune suppressive effects of regulatory T cells (T-reg) in controlling inflammation and preventing autoimmunity is well-established. Maternal immune tolerance to fetus is also integral to successful pregnancy. Vitamin D deficiency is quite common in pregnancy which has been associated with adverse outcomes. Cyprian et al. reviewed the consequences of vitamin D deficiency during pregnancy and detailed the role of T-regs in mitigating pregnancy loss and adverse events such as preeclampsia. Further, Vitamin D is shown to play an important role in modulating graft-vs-host disease (GvHD) by balancing immune responses. Flamann et al. described the role of vitamin D in inducing immune suppressive T regs and balancing immune responses in allogeneic hematopoietic stem cell transplantation. The immune modulatory effects of vitamin D increased immune tolerance and enhanced anti-tumor activity.

In cases of autoimmune diseases, low vitamin D levels are associated with increased B cell proliferation and autoantibody production. Low vitamin D levels have also been associated with the incidence of autoimmune diseases such as Rheumatoid Arthritis (RA), Multiple Sclerosis (MS), and Systemic Lupus Erythematosus (SLE) (15). A systematic review by Islam et al. on the immunomodulatory effects of diet and nutrients in Systemic Lupus Erythematosus (SLE) presented the beneficial effect of vitamin D supplementation such as alleviating fatigue. Meta-analysis studies showed that SLE patients have significantly lower vitamin D levels compared to healthy participants. In human and animal studies of SLE, vitamin D is shown to inhibit cytokines such as IL-10, IL-17, IFN-γ, and modulate cellular proliferation of B-cells, Th1, Th17, CD4+ T cells. Therefore, vitamin D exerts various immune modulatory effects during autoimmune diseases. Another example of autoimmune diseases is Type 1 diabetes mellitus (T1DM) where insulin producing pancreatic β-cells are attacked by T cell-mediated autoimmunity (12, 16). Stem cell transplantation has emerged as regenerative treatment for T1DM as it possess immune suppressive potential. A clinical trial (NCT03920397) conducted by Araujo et al. on the effect of vitamin D treatment with allogenic adipose tissue-derived stromal/stem cells (ASCs) in 13 patients with recent onset of T1DM. One group received ASC and vitamin D (cholecalciferol 2,000 UI/day) for 3 months while the second group received standard insulin therapy. They observed that allogenic ASC and vitamin D therapy lead to reduced insulin requirement and more stable C peptide.

Low vitamin D status has been shown to be a risk factor for infectious diseases (17, 18). During infections such as Tuberculosis (TB), vitamin D induces the expression of cathelicidin (LL-37), a host defense peptide that enhances the bactericidal activity of immune cells like macrophages, thereby limiting the growth of mycobacteria that causes TB (18). Furthermore, LL-37 exerts anti-inflammatory effects via its ability to neutralize bacterial molecules like endotoxins and capsular polysaccharides that activate TLR signaling pathways, consequently inhibiting the release of pro-inflammatory mediators from macrophages. In this special topic several research papers investigated the role of vitamin D in infection outcomes. Muvva et al. investigated the effect of vitamin D on controlling intracellular *Mycobacterium tuberculosis* (Mtb) infection in human monocytes-derived macrophages. They showed that vitamin D treatment resulted in an appropriate macrophage polarization M1/M2 phenotype with enhanced expression of antimicrobial LL-37 and anti-inflammatory cytokine IL-10 but reduced expression of the immune suppressive enzyme IDO. This *in vitro* study provides evidence to the immune modulatory effects of vitamin D in controlling Mtb infection. Similarly, LL-37 is shown to control Leishmania parasitic infections in human. Crauwels et al. observed increased expression of LL-37 in skin biopsies from cutaneous leishmaniasis. They demonstrated that recombinant LL-37 reduced Leishmania parasite viability in a dose-dependent manner. Accordingly, they investigated the effect of vitamin D treatment on human monocytic-derived macrophages and documented that LL-37 mediated Leishmania restriction in macrophages. Vitamin D role in controlling infection is also demonstrated in acute enterocolitis. Mousavi et al. used an animal model to investigate the beneficial effects of vitamin D on preserving intestinal barrier and exerting anti-pathogenic effects during *Campylobacter jejuni* infection. They reported that preclinical administration of vitamin D ameliorated acute campylobacteriosis in mice model by reducing bacterial colonization and translocation, restoring intestinal epithelial cells regeneration and dampening inflammatory responses.

Vitamin D deficiency increases the risk of bacterial and viral infections (17). The immune modulatory effects of vitamin D during bacterial infections seem to vary from those effects during viral infections. In this regards, Anderson et al. observed the differential effects of vitamin D on induced inflammatory responses in primary human peripheral mononuclear cells (PBMC) during bacterial pneumococcal infection alone, respiratory syncytial viral (RSV) infection or co-infection. They showed that vitamin D exerted anti-inflammatory effects by reducing Th17 inflammatory cell expression and cytokines during pneumococcal infection alone and RSV alone but not during co-infection with RSV.

Genetic predisposition plays a role in host susceptibility to infections (19). Similarly, polymorphism in genes involved in vitamin D metabolism and function such as VDR impacts host susceptibility to infections. Pepineli et al. evaluated four genetic variants of VDR in the Leprosy immune pathogenesis in Brazil. They investigated a cohort of 404 leprosy patients in comparison to 432 control individuals without disease. Although no association is identified in VDR polymorphism genetic frequency between patients with active clinical leprosy and controls, they observed bAt haplotype to confer protection from leprosy. Taken together, data from original research paper, clinical trial, systematic analysis, and reviews included herein provide compelling evidence to the immune modulatory effects of vitamin D. Therefore, vitamin D deficiency should be treated to gain the extra skeletal beneficial effects on the immune system in health and disease.

## Author Contributions

SZ wrote the editorial. EL and AB reveiwed the editorial. All authors contributed to the article and approved the submitted version.

## Conflict of Interest

The authors declare that the research was conducted in the absence of any commercial or financial relationships that could be construed as a potential conflict of interest.
